# Suppression of iASPP-dependent aggressiveness in cervical cancer through reversal of methylation silencing of microRNA-124

**DOI:** 10.1038/srep35480

**Published:** 2016-10-21

**Authors:** Peixin Dong, Ying Xiong, Hidemichi Watari, Sharon JB Hanley, Yosuke Konno, Kei Ihira, Fumihiko Suzuki, Takahiro Yamada, Masataka Kudo, Junming Yue, Noriaki Sakuragi

**Affiliations:** 1 Department of Women’s Health Educational System, Hokkaido University School of Medicine, Hokkaido University, N15, W7, Sapporo 0608638, Japan; 2 Department of Gynecology, State Key Laboratory of Oncology in South China, Sun Yat-sen University Cancer Center, Guangzhou 510060, P. R. China; 3 Department of Gynecology, Hokkaido University School of Medicine, Hokkaido University, N15, W7, Sapporo 0608638, Japan; 4 Department of Obstetrics and Gynecology, Tohoku University, Sendai 9808574, Japan; 5 Department of Pathology and Laboratory Medicine, University of Tennessee Health Science Center, Memphis, TN, USA; 6 Center for Cancer Research, University of Tennessee Health Science Center, Memphis, TN, USA

## Abstract

Derepression of wild-type p53 by suppressing its negative inhibitor iASPP (Inhibitor of apoptosis-stimulating protein of p53) represents a potential therapeutic option for cervical cancer (CC). Here, we reported a novel functional significance of iASPP upregulation in cervical tumorigenesis: iASPP acts as a key promoter of CC cell proliferation, epithelial-mesenchymal transition, invasion and cancer stemness, by interacting with p53 to suppress p53-mediated transcription of target genes and reducing p53-responsive microRNA-34a levels. Moreover, we demonstrate that miR-124, directly targeting iASPP, reduces expression of iASPP and attenuates CC cell growth and invasiveness. Low miR-124 expression is inversely correlated with increased expression of iASPP mRNA in CC tissues. In a cohort of 40 patients with CC, the low miR-124 expression was correlated with poor 5-year overall survival (*P* = 0.0002) and shorter disease-free survival 5-year (*P* = 0006). Treatment with the DNA methyltransferase inhibitor Zebularine increases miR-124 expression and retards CC cell growth and invasion with minimal toxicity to normal cells. Even at a non-toxic concentration, Zebularine was effective in suppressing CC cell invasion and migration. Altogether, the restoration of miR-124 reduces iASPP expression and leads to p53-dependent tumor suppression, suggesting a therapeutic strategy to treat iASPP-associated CC.

Cervical cancer (CC) is a common malignancy in women worldwide (>250,000 deaths/year), and the 5-year survival rate of patients with advanced disease remains as low as 40%^1^. Most CCs express wild-type (WT) p53, and the disruption of WT p53 function (rather than the presence of p53 mutations) plays a crucial role in CC progression^2^. E6 proteins encoded by the high-risk HPV types, which cause approximately 70% of all CCs, impair p53 function by stimulating ubiquitin-mediated degradation of p53^3^ or by interfering with p53-DNA binding activity^4^. In addition, some other genetic mechanisms for inactivating p53 function in human tumors have been proposed^5^. Inhibitor of apoptosis-stimulating protein of p53 (iASPP) binds directly to the DNA-binding domain of p53 and inhibits its functions^6^. The overexpression of iASPP is a common event seen in many human cancers^5^ and is associated with advanced stage, lymph node metastasis, chemoresistance, radioresistance and decreased survival of CC patients^7^. Whether targeting iASPP can restore p53 tumor suppressor functions and how epigenetic mechanisms, such as aberrant microRNA (miRNA) expression, regulate iASPP expression in CC remains unclear.

In this study, we demonstrate that iASPP not only inhibits the DNA-binding and transcriptional functions of p53 on the promoters of its downstream genes but also represses the expression of p53-responsive miR-34a, leading to the promotion of proliferation, epithelial-mesenchymal transition (EMT), invasion and cancer stem cell-like properties of CC cells. Furthermore, we show that iASPP is targeted by miR-124, which is a key tumor suppressor in CC cells and is highly induced after Zebularine treatment. These data point to miR-124 as a potential therapeutic target in iASPP-overexpressing CC.

## Results

### iASPP promotes proliferation, EMT, invasiveness and cancer stem cell phenotypes of CC cells

**Figure 1:**
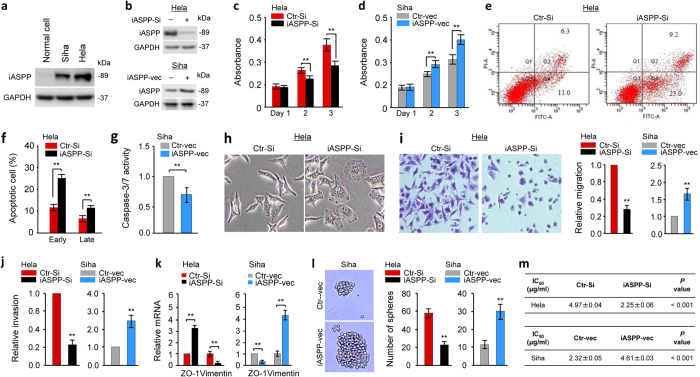
iASPP promotes proliferation, EMT, invasiveness and cancer stem cell phenotypes of CC cells. (**a**) Immunoblot of the iASPP protein in CC cell lines and a normal endometrial epithelial cell line. (**b**) Western blot analysis of the iASPP protein in CC cells after transfection with iASPP siRNA (iASPP-Si), iASPP expression vector (iASPP-vec) or corresponding negative controls. (**c,d**) CC cells were transfected with iASPP-Si, control siRNA (Ctr-Si), iASPP-vec or empty control vector (Ctr-vec), and cell proliferation was measured using a cell counting kit-8 assay at the selected time points. (**e**) Cell apoptosis was measured by flow cytometry analysis of Annexin V-FITC double-labeled Hela cells transfected with iASPP-Si or Ctr-Si. (**f**) The early apoptotic and late apoptotic rates of Hela cells transfected with iASPP-Si or Ctr-Si. (**g**) Caspase-3/7 activities in Siha cells expressing either Ctr-vec or iASPP-vec. (**h**) Phase-contrast microscopy shows the morphology of Hela cells transfected with Ctr-Si or iASPP-Si. (**i**) Left: Representative image of cell migration of Hela cells in cell migration assay. Right: Relative migration of Hela cells after knockdown and Siha cells after overexpression of iASPP, respectively. (**j**) Relative invasion of Hela cells after knockdown and Siha cells after overexpression of iASPP, respectively. (**k**) The qPCR analysis of *ZO-1* and *Vimentin* expression in Hela (left) and Siha (right) cells, transfected as indicated. (**l**) Left: Representative images of spheres formed from Siha cells transfected with Ctr-vec or iASPP-vec. Right: The number of spheres formed by CC cells transfected with either iASPP-Si or iASPP-vec as indicated. (**m**) CC cells transfected as indicated were treated with cisplatin for 48-hours and then cell viability was analyzed using a cell counting kit-8 assay. The data are shown as the mean ± SEM; n = 3; **P  <  0.01.

To directly address the role of iASPP in CC cells, we first determined whether CC cells are characterized by increased iASPP protein levels. As shown in Fig. 1a, the iASPP antibody (sc-81297) used in this study specifically detected endogenous iASPP. In the human CC cell lines Hela and Siha, we observed high levels of iASPP, compared with normal endometrial epithelial cells, where expression of iASPP was extremely weak. These data imply that iASPP overexpression might accelerate tumorigenesis and metastasis of CC.

Using short-interfering RNA (siRNA) targeting iASPP mRNA (iASPP-Si), we assessed the effects of iASPP depletion on the proliferation of Hela cells, which express relatively higher levels of iASPP protein (Fig. 1b). Downregulation of iASPP significantly reduced the proliferation rates of Hela cells, as measured by a cell counting kit-8 assay (Fig. 1c). Conversely, transient overexpression of iASPP using an iASPP expression vector (iASPP-vec) in Siha cells, which has relatively lower levels of iASPP than Hela cells, caused a significant promotion of cell proliferation (Fig. 1d). To explore the mechanism of cell growth retardation in more detail, we determined the effects of iASPP knockdown on the cell apoptosis in Hela cells using flow cytometry with Annexin V staining (Fig. 1e). Our results demonstrated an significant increase in the numbers of both early and late apoptotic cells in iASPP-Si-transfected Hela cells compared with those observed in the control siRNA (Ctr-Si)-transfected cells (Fig. 1f). Consistent with these data, the overexpression of iASPP in Siha cells inhibited the activity of caspase-3 and caspase-7 (Fig. 1g), suggesting that iASPP promotes cell proliferation and inhibits apoptosis in CC cells.

To determine whether iASPP affects invasive phenotypes in CC cells, we examined the effect of either iASPP knockdown or overexpression on cell morphology, invasion, migration and the expression of EMT-related markers. The depletion of iASPP in Hela cells induced a more epithelial-like phenotype (Fig. 1h); however, ectopic expression of iASPP induced mesenchymal-like morphological features in Siha cells (data not shown). In Matrigel-coated transwell experiments, we found that the downregulation of iASPP significantly decreased the migration and invasion potential of Hela cells (Fig. 1i,j). We also observed that overexpression of iASPP increased the migration and invasion of Siha cells (Fig. 1i,j). Quantitative PCR (qPCR) analysis of the epithelial marker *ZO-1* and the mesenchymal marker *Vimentin* revealed that knockdown of iASPP increased *ZO-1* and decreased *Vimentin* expression (Fig. 1k). Conversely, elevated iASPP expression downregulated *ZO-1* as well as upregulated *Vimentin* in Siha cells (Fig. 1k). To examine if iASPP affects cancer stem cell function, we first examined its ability to influence self-renewal *in vitro* using the sphere formation assay. The iASPP-Si-transfected Hela cells generated smaller and fewer spheres as compared with Ctr-Si-transfected cells (Fig. 1l). In agreement with this observation, iASPP-vec-expressing Siha cells showed an increase in sphere-forming ability relative to cells transfected with the control vector (Ctr-vec) (Fig. 1l). We further investigated the role of iASPP in chemoresistance using a cell counting kit-8 assay. As shown in Fig. 1m, Hela cells transfected with iASPP-Si exhibited a lower resistance and IC50 to cisplatin than those transfected with Ctr-Si. In contrast, the upregulation of iASPP in Siha cells increased the resistance and IC50 value of cisplatin. Altogether, these results show that iASPP promotes a more proliferative, invasive and cancer stem cell-like phenotype of CC cells.

### iASPP interacts with p53 to suppress its transcriptional activities toward target genes, and represses the expression of p53-responsive miR-34a

**Figure 2:**
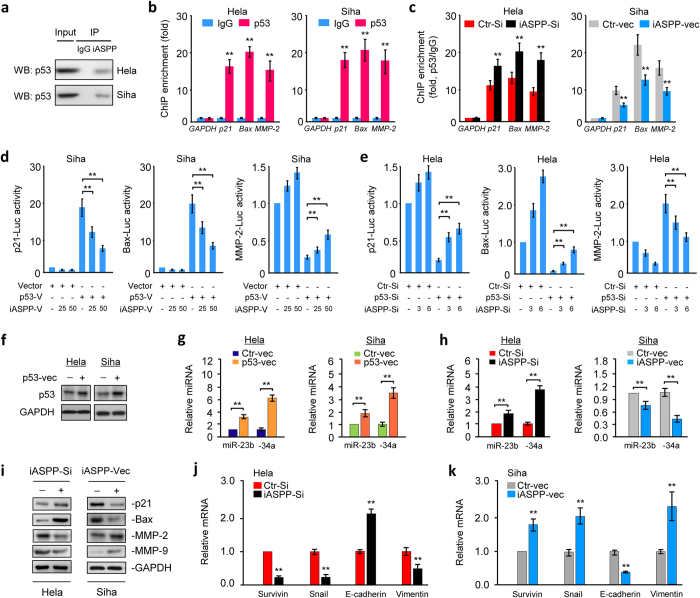
iASPP interacts with p53 to suppress its transcriptional activities toward target genes, and represses the expression of p53-responsive miR-34a. (**a**) Co-immunoprecipitation (Co-IP) assays reveal associations between endogenous iASPP and p53 in CC cells. Cell lysates were labeled as input. IgG was used as the control. (**b**) Chromatin immunoprecipitation (ChIP)-qPCR assays confirmed the occupancy of p53 at *p21*, *Bax* and *MMP-2* loci, but not at the *GAPDH* promoter. (**c**) ChIP-qPCR assays. *p21*, *Bax* and *MMP-2* promoter regions containing p53-binding sites were quantified by qPCR in CC cells after overexpression or knockdown of iASPP. Fold change of enrichment was determined relative to IgG controls and *GAPDH*. (**d**) Siha cells were transfected with *p21*-, *Bax*-, or *MMP-2*-promoter luciferase plasmids, together with (or without) p53 expression vector, in the presence (or absence) of increasing amounts of iASPP expression vector. Luciferase activities were corrected for transfection efficiency by normalization to Renilla luciferase activity. (**e**) Hela cells were transfected with the indicated promoter luciferase plasmids, together with (or without) p53 siRNA, in the presence (or absence) of increasing amounts of iASPP-Si. Relative reporter activities were assessed. (**f**) Protein expression of p53 in CC cells transfected with control or p53 expression vector. qPCR analysis of miR-23b and miR-34a expression in CC cells expressing either the control or p53 vector (**g**), or in CC cells after overexpression or knockdown of iASPP (**h**). (**i**) Immunoblot of the indicated proteins in CC cells after knockdown or overexpression of iASPP. (**j**) qPCR analysis of the indicated mRNAs in Hela cells following iASPP knockdown, or in Siha cells after iASPP overexpression. The data are shown as the mean ± SEM; n = 3; **P  <  0.01.

To define whether iASPP can directly interact with p53 in CC cells, we examined the binding between endogenous iASPP and p53 using a co-immunoprecipitation assay. Indeed, in Hela and Siha cells, p53 was co-immunoprecipitated with iASPP-specific antibody but not with a control antibody (Fig. 2a), suggesting that iASPP and p53 bind to each other in CC cells. Then, we asked whether the binding of iASPP to p53 modulates the DNA-binding functions of p53 on promoter regions of the known p53 target genes *p21*, *Bax* and *MMP-2*^8^,^9^,^10^, by performing chromatin immunoprecipitation (ChIP)-qPCR assays. Our results demonstrated that anti-p53 antibody, but not nonrelated immunoglobulin G (IgG), pulled down the promoters of *p21*, *Bax* and *MMP-2* but not the promoter region of *GAPDH* gene^11^ in CC cells (Fig. 2b). To investigate if iASPP functionally regulates p53 recruitment to target regulatory regions, we knocked down or overexpressed iASPP and measured the binding of p53 to the regulatory regions of the target genes. The ChIP-qPCR assay results showed that the knockdown of iASPP significantly promoted p53 recruitment to the promoters of *p21*, *Bax* and *MMP-2*, whereas overexpression of iASPP diminished p53 recruitment to its target promoters (Fig. 2c), revealing that iASPP prevents p53 recruitment to its gene promoters in CC cells. To further explore the role of iASPP in regulating p53 transcriptional functions, we co-transfected luciferase constructs containing one of *p21*^12^, *Bax*^12^ and *MMP-2*^13^ promoters in combinations with p53 expression vector, p53 siRNA, iASPP-vec or iASPP-Si into Siha and Hela cells. The reporter gene assays suggest that the introduction of p53 activated *p21* and *Bax* promoter activities but suppressed *MMP-2* promoter activity. In accordance with these results, silencing of p53 significantly decreased *p21* and *Bax* promoter-driven luciferase expression but enhanced *MMP-2* activity (Fig. 2d,e). We found that co-transfection of iASPP-vec reversed p53-mediated transactivation of the *p21* and *Bax* promoters and p53-mediated transrepression of the *MMP-2* promoter in a dose-dependent manner (Fig. 2d). In agreement with the above data, transfection with iASPP-Si can revert the p53 siRNA-induced transcriptional effects (Fig. 2e and Supplementary Fig. S1), suggesting that iASPP suppresses the transcriptional activities of p53 on its responsive target promoters.

Moreover, p53 induces the expression of tumor suppressor miRNAs such as miR-23b^14^ and miR-34a^15^ via direct transactivation of these miRNAs. Hela and Siha cells transiently transfected with a p53 expression vector demonstrated elevated p53 levels (Fig. 2f) and increased miR-23b and miR-34a expression (Fig. 2g) as compared to cells transfected with the control vector. Then we examined whether iASPP modulates the expression of these miRNAs using qPCR analysis. In Hela cells, the downregulation of iASPP resulted in enhanced miR-23b and miR-34a levels; however, the overexpression of iASPP in Siha cells inhibited their expression (Fig. 2h). Considering that miR-34a levels were upregulated more significantly than miR-23b after iASPP knockdown, we sought to determine whether miR-34a alters CC cell invasion and growth. Ectopic miR-34a expression with miR-34a mimic decreased the invasion and proliferation of Hela and Siha cells (Supplementary Fig. S2a,b) and led to the upregulation of E-cadherin and downregulation of the known miR-34a targets Survivin and Snail as well as Vimentin (Supplementary Fig. S2c,d), demonstrating that iASPP might exert its oncogenic effects through the regulation of tumor suppressive mR-34a in CC cells. Using immunoblots and qPCR, we found that iASPP knockdown in Hela cells increased the levels of p21, Bax and *E-cadherin* and reduced the expression of MMP-2, MMP-9 (another transcriptional target of p53^16^), *Survivin*, *Snail* and *Vimentin* (Fig. 2i–k). Forced expression of iASPP demonstrates the reverse effect in Siha cells. Taken together, these data show that iASPP promotes cell proliferation, EMT, invasion and cancer stem cell-like properties of CC cells, by directly binding p53 to inhibit p53 DNA-binding and transcriptional functions on downstream gene promoters and by repressing p53-responsive miR-34a.

### Oncogene *iASPP* is a direct target of miR-124 in CC cells

**Figure 3:**
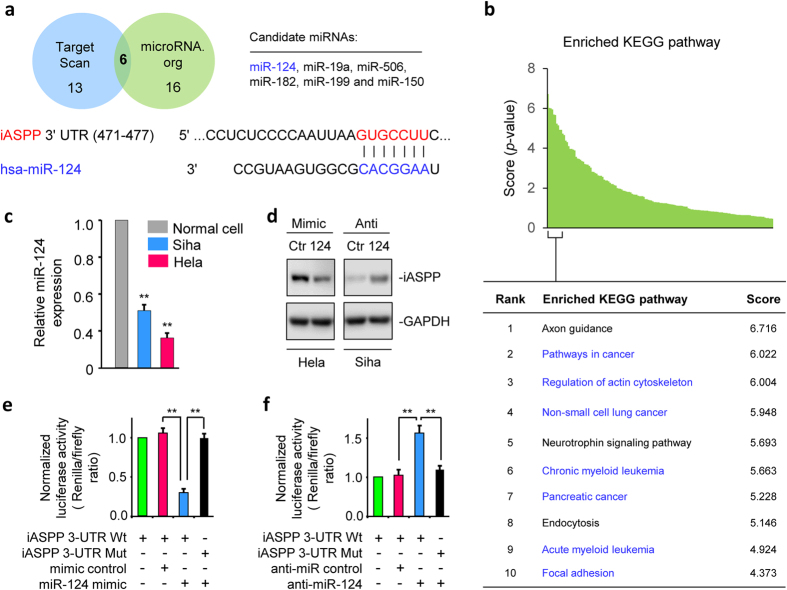
Oncogene *iASPP* is a direct target of miR-124 in CC cells. (**a**) Summary of the number of miRNAs that were predicted to bind to the 3′-UTR of *iASPP* by TargetScan and microRNA.org (upper panel). The 6 predicted miRNAs were common to these two algorithms. Schematic representation of the 3′-UTR of *iASPP* with the predicted target site for miR-124 (lower panel). (**b**) *In silico* prediction and molecular pathway enrichment analysis was performed on predicted target genes regulated by miR-124. The top 10 ranking KEGG pathways are listed. (**c**) qPCR analysis of miR-124 expression in CC cell lines and a normal cell line. The results are presented as the fold-change in expression compared with normal cell. (**d**) Expression of the iASPP mRNA and protein in CC cells after the transient overexpression or knockdown of miR-124. Hela (**e**) and Siha (**f**) cells were cotransfected with reporter plasmids containing wild-type *iASPP* or a mutant *iASPP* 3′-UTR together with a miR-124 mimic, miR-124 inhibitor, or respective negative controls. The relative luciferase activity was assayed. Data are shown as mean ± SEM; n = 3; **P  <  0.01.

Using two miR algorithms (TargetScan and microRNA.org), we identified 6 miRNAs that were consistently predicted to interact with the 3′-UTR of *iASPP* mRNA (Fig. 3a). Our pathway analysis using the miRSystem algorithm^17^ identified miR-124 as an interest candidate, because potential miR-124 targets were mostly associated with and enriched in well-known oncogenic and metastatic KEGG pathways (Fig. 3b). Our qPCR assays revealed significantly lower miR-124 in CC cells that express relatively higher levels of iASPP when compared to normal cells (Fig. 3c). After the introduction of miR-124 mimic iASPP protein levels decreased (Fig. 3d). In contrast, the inhibition of miR-124 by using an antisense miRNA inhibitor (anti-124) increased iASPP levels (Fig. 3d). To analyze whether miR-124 directly represses iASPP protein translation by binding to the 3′-UTR, we cotransfected a reporter construct containing *iASPP* 3′-UTR with miR-124 mimic or anti-miR-124 into CC cells. In dual luciferase assays, miR-124 mimic inhibited iASPP luciferase activity in Hela cells (Fig. 3e), whereas anti-miR-124 increased iASPP luciferase activity in Siha cells (Fig. 3f). Mutation in miR-124-binding site abrogated these regulatory effects of miR-124 (Fig. 3e,f). Together, these results implicate miR-124 as a direct regulator of *iASPP* mRNA.

### MiR-124 suppresses the metastatic phenotypes of CC cells, possibly through suppressing its target iASPP

**Figure 4:**
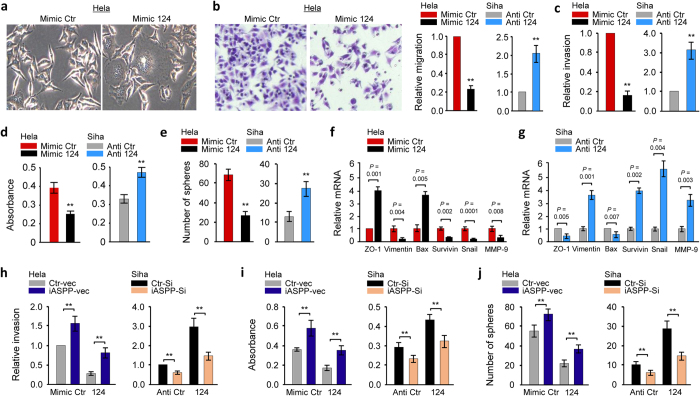
MiR-124 suppresses metastatic phenotypes of CC cells by suppressing its target iASPP. (**a**) Morphology of Hela cells transfected with miR-124 mimic (Mimic 124) or negative control mimic (Mimic Ctr). (**b**) Left: Representative image of Hela cell migration in cell migration assay. Right: Relative migration of Hela cells after transient overexpression or Siha cells after knockdown of miR-124. Invasion (**c**), proliferation (**d**), sphere formation (**e**) and qPCR analysis of indicated mRNAs (**f**,**g**) in CC cells after transient overexpression or knockdown of miR-124. Mimic 124 or mimic Ctr was transfected into Hela cells, together with (or without) *iASPP* cDNA vector lacking the 3′-UTR region. Anti-miR-124 inhibitor or anti-miRNA control was transfected into Siha cells, together with (or without) iASPP-Si. The cells were assayed for cell invasion (**h**), proliferation (**i),** and sphere formation (**j**). Data are shown as mean ± SEM; n = 3; **P  <  0.01.

To determine whether miR-124 can inhibit CC development, we manipulated miR-124 levels in CC cells and performed cell functional assays. Hela cells transfected with miR-124 mimic exhibited round and enlarged morphological changes (Fig. 4a) and less motility and invasion as well as weaker proliferative and sphere-forming abilities than cells transfected with the mimic control (Fig. 4b–e). Inhibition of miR-124 by means of anti-124 promoted these malignant features of Siha cells (Fig. 4b–e). Similarly, overexpression of miR-124 mimic in another CC cell line CaSki confirmed its inhibitory roles in CC invasion and proliferation (data not shown). We observed an upregulation of *ZO-1* and *Bax*, and downregulation of *Survivin*, *Snail* and *MMP-9* in miR-124 mimic-transfected Hela cells (Fig. 4f). The reversed effects were observed in Siha cells when miR-124 was knocked down (Fig. 4g). These data support that miR-124 negatively regulates the invasive and stem cell-like properties of CC cells.

Furthermore, we ectopically expressed the *iASPP* cDNA lacking the 3′-UTR in Hela cells transfected with the miR-124 or control mimic. The expression of iASPP could relieve miR-124-mediated inhibition of invasion, proliferation and sphere formation (Fig. 4h–j). Next, we attenuated the expression of iASPP in Siha cells with siRNA together with inhibition of miR-124, and found that suppression of iASPP largely inhibited the ability to invade, proliferate and to generate spheres induced by miR-124 inhibition (Fig. 4h–j). Together, these data indicate that miR-124 suppresses the metastatic phenotypes of CC cells, possibly through suppressing its target iASPP and subsequent activation of p53 signaling in CC cells.

### Low miR-124 expression is associated with a worse outcome and inversely correlated with iASPP overexpression in CCs

**Figure 5:**
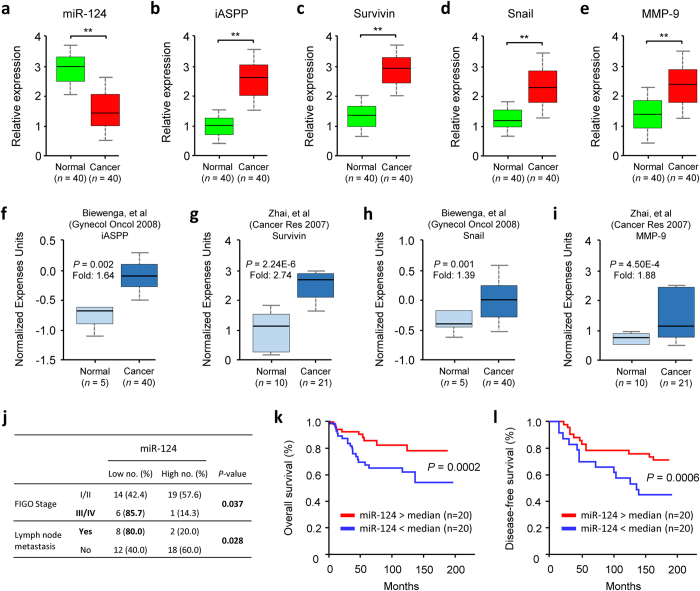
Low miR-124 expression is associated with worse outcome and inversely correlated with iASPP overexpression in CCs. The expression levels of miR-124 (**a**), *iASPP* (**b**), *Survivin* (**c**), *Snail* (**d**) and *MMP-9* (**e**) were assessed by qPCR analysis of 40 paired cancerous and normal tissue samples from CC patients. Data are shown as mean ± SEM; n = 3; **P  <  0.01. Analysis of *iASPP* (**f**), *Survivin* (**g**), *Snail* (**h**) and *MMP-9* (**i**) mRNA expression using microarray (Oncomine) on normal cervical versus CC patient data sets. (**j**) Correlations between miR-124 expression and clinicopathological features of patients with CC. Kaplan-Meier analysis of overall (**k**) and disease-free survival (**l**) in 40 CC patients according to high and low miR-124 expression.

In a cohort of 40 CC patients, we showed that CC tissues have lower miR-124 levels and higher *iASPP*, *Survivin*, *Snail* and *MMP-9* mRNA expression compared with adjacent normal tissues (Fig. 5a–e). Furthermore, Oncomine analysis of published expression microarray data revealed the upregulation of *iASPP*, *Survivin*, *Snail* and *MMP-9* transcripts in CCs compared with normal cervical tissues (Fig. 5f–i). To explore the clinical significance of the loss of miR-124 expression in CC, we divided 40 CCs into two groups with higher (n = 20) or lower miRNA expression (n = 20) using the median value as a cutoff. Having lower levels of miR-124 was significantly associated with advanced tumor stage and lymph node metastasis (Fig. 5j). The Kaplan–Meier survival analyses showed that CC patients with lower miR-124 expression had significantly shorter 5-year overall survival (*P* = 0.0002) and 5-year disease-free survival rates (*P* = 0.0006) than those with higher miR-124 expression (Fig. 5k,l). Collectively, these data support an inverse relationship between miR-124 and iASPP expression, and suggest the potential use of miR-124 as a prognostic biomarker and a therapeutic target in CC.

### Reversal of methylation silencing of miR-124 via Zebularine reduces iASPP expression and retards CC cell growth and invasion

**Figure 6:**
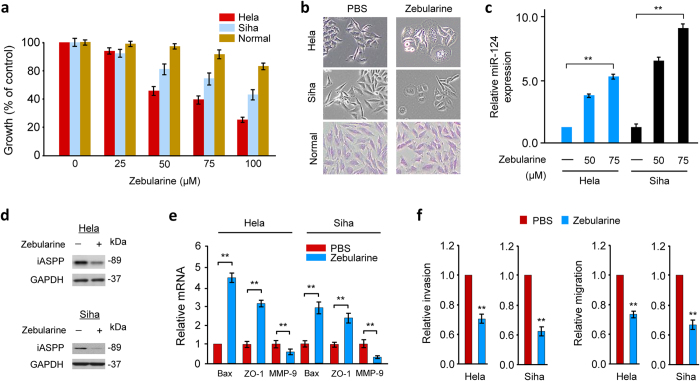
Reversal of methylation silencing of miR-124 via Zebularine reduces iASPP expression and retards CC cell growth and invasion. (**a**) CC cells and normal endometrial epithelial cell were treated with Zebularine or PBS for 72 hours, and cell growth was determined using cell counting kit-8 assay. (**b**) Morphology of CC cells and normal cell treated with Zebularine (50 μM) or PBS for 72 hours. (**c**) qPCR analysis of miR-124 expression in CC cells treated with Zebularine (50, 75 μM) or PBS. Protein expression of iASPP (**d**) and expression of indicated mRNAs (**e**) in CC cells after treatment with Zebularine (50 μM) or PBS. (**f**) Invasion (left) and migration (right) of CC cells treated with Zebularine (25 μM) or PBS.

Because miR-124 is silenced by DNA methylation in CC cells^18^, we examined whether reactivation of miR-124 with a DNA methyltransferase inhibitor Zebularine could affect iASPP expression and CC cell growth and invasion. First, we treated CC cells and normal cells with Zebularine. Cell counting kit-8 assays demonstrated that Zebularine exhibited efficient anti-proliferative activity in Hela and Siha cells with IC50 values of 50 μM to 100 μM, respectively (Fig. 6a). Interestingly, Zebularine displayed no evident toxicity on normal cells (Fig. 6a). As shown in Fig. 6b, CC cells treated with Zebularine changed their mesenchymal morphology and acquired a more epithelial morphology. However, Zebularine did not affect normal cell morphology (Fig. 6b). Treatment with Zebularine increased miR-124 levels in a dose-dependent manner (Fig. 6c) and also decreased iASPP (Fig. 6d) expression in CC cells. This was accompanied by increased levels of *Bax* and *ZO-1* mRNAs and by reduced *MMP-9* mRNA levels (Fig. 6e). Even at a non-toxic concentration (25 μM), Zebularine can significantly inhibit the invasion and migration of CC cells (Fig. 6f). Collectively, these data support that re-expression of miR-124 by treatment with Zebularine can reduce iASPP expression, leading to p53-dependent tumor suppression in CC cells.

## Discussion

**Figure 7:**
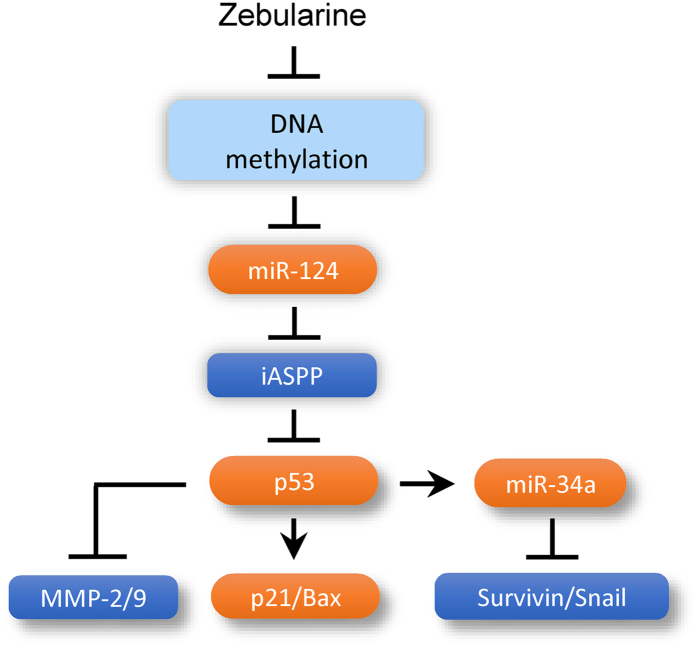
A schematic model of the regulation of CC proliferation, invasion, stemness and metastasis by miR-124-iASPP-p53 regulatory axis. The expression of miR-124 is silenced by DNA methylation in CC. The restoration of miR-124 via Zebularine reduces the expression of iASPP (a direct suppressor of p53), thereby activating the p53 signaling network and suppressing tumor growth, EMT, invasion, cancer stemness and metastasis.

Our study establishes that iASPP plays a powerful role in controlling multiple malignant properties of human CC including proliferation, apoptosis, migration, invasion, self-renewal and chemotherapy resistance. Mechanistically, iASPP promotes these oncogenic features by interacting with p53 and inhibiting its ability to regulate transcription of target genes and by repressing the expression of p53-responsive miR-34a. We further showed that miR-124, whose expression can be restored by a safe methyltransferase inhibitor Zebularine, functions as a pivotal tumor suppressor in CC by repressing the expression of iASPP and subsequently activating the p53 signaling network (Fig. 7).

In tumors that retain a WT p53 gene, p53 function is generally impaired^5^. Many efforts have been made to reactivate p53 functions in human cancers including CC, by suppressing the expression of its inhibitor HPV E6^19^ or by interfering with the binding between inhibitory proteins and p53^20^,^21^,^22^. In our experiments, the depletion of a novel p53 suppressor iASPP is capable of enhancing p53 binding to downstream target gene promoters, resulting in the functional re-activation of p53 network and significant tumor repression. Our findings suggest that the downregulation of iASPP might represent an attractive therapeutic approach for CC.

MiR-124 has been observed to show tumor suppressor properties and to be downregulated in a broad range of tumor types including CC^18^,^23^,^24^. In agreement with previous studies on CC^18^,^24^, we confirmed that miR-124 reduces CC cell proliferation, migration and invasion. More importantly, our observations suggest that miR-124 serves as a key suppressor of EMT and cancer stemness. Overexpression of miR-124 counteracts cancer stem cell phenotypes, at least through downregulating iASPP levels. Thus, the restoration of miR-124 might prevent prevents CC initiation and progression by eliminating EMT-phenotypic cells or cancer stem cells, which are believed to be the cause of tumor maintenance, tumor metastasis and recurrence.

In contrast with some DNA methylation inhibitors (such as 5-Azacytidine and 5-aza-2′-deoxycytidine) that are relatively unstable and toxic, Zebularine exhibits chemical stability and preferentially targets cancer cells with minimal toxicity to normal cells, via p53-dependent mechanisms^25^,^26^. Here we found that the addition of Zebularine inhibited the growth of CC cells but not of a normal cell line. Even at a non-toxic concentration, this drug was still effective in suppressing CC cell invasion and migration, indicating that Zebularine can block CC cell invasion and motility independent of its inhibitory effects on cell proliferation. Our findings are consistent with the reported roles of Zebularine in inhibiting proliferation of lung cancer cells by inducing demethylation of the tumor suppressor ABCB4^27^, and highlight the potential translational application of Zebularine for the safe and selective treatment of CC. The detailed effects of Zebularine *in vivo* or on gene/miRNA expression in CC requires further investigation.

In conclusion, the restoration of miR-124 via Zebularine inhibits iASPP expression and induces p53-dependent suppression in CC.

## Methods

All methods were performed in accordance with relevant guidelines and regulations.

### Human CC specimens

40 pairs of primary CC specimens and adjacent non-tumor cervical tissues were collected following informed consent and approval of the ethical committee at the Cancer Center, Sun Yat-Sen University in China. Samples were snap-frozen and stored in liquid nitrogen until the RNA was extracted.

### Cell culture, transfection and drug treatment

Human CC cell lines Hela and Siha (ATCC), which express WT p53, and the immortalized human normal endometrial epithelial cell line^28^ were maintained in DMEM/F12 medium (Sigma-Aldrich, St. Louis, MO) supplemented with 10% fetal bovine serum (FBS; Invitrogen, Carlsbad, CA). Human CC cell line CaSki was kindly provided by Dr. Fumihiko Suzuki (Tohoku University, Sendai, Japan), and maintained in RPMI-1640 medium (Sigma-Aldrich, St. Louis, MO) supplemented with 10% FBS. The miRNA mimics, anti-miRNA inhibitor, iASPP and p53 siRNA, and respective negative controls were purchased from Ambion (TX). The miRNAs (30 nM) and siRNAs (3 or 6 nM) were transiently transfected using Lipofectamine 2000 reagent (Invitrogen, CA). The iASPP expression vector, p53 expression vector and empty control vector (OriGene, MD) were transiently transfected using the Lipofectamine Plus reagent (Invitrogen, CA, USA). The control vector and a human iASPP cDNA vector lacking the 3′-UTR sequence were obtained from OriGene (MD). The cells were treated with PBS or Zebularine (25–100 μM, Stem Cell Technologies, Vancouver, BC) for 72 hours. Fresh medium containing Zebularine was added daily.

### RNA isolation and qPCR

Total RNA was isolated using TRIzol (Invitrogen, CA) according to the manufacturers protocol. For the mRNA and miRNA analysis, cDNA was generated from 100 ng of total RNA using a PrimeScript RT reagent kit (Takara, Japan). The mRNA levels were quantified using Takara SYBR Premix Ex Taq II (Takara, Japan). Primers specific to *iASPP*^29^ and *GAPDH*^30^ have been previously described. Primers for *ZO-1*, *Vimentin*, *Bax*, *Survivin*, *Snail*, *MMP-2* and *MMP-9* were obtained from the PrimerBank database (http://pga.mgh.harvard.edu/primerbank/). The miRNA expression was detected by using the NCode miRNA qRT-PCR kit (Invitrogen, CA), forward primers (the exact sequences of the mature miRNA genes) and a universal reverse primer supplied by the manufacturer. All mRNA and miRNA quantification data were normalized to *GAPDH* and U6 small nuclear RNA^31^, respectively. Results were represented as fold change using the 2^−ΔΔCt^ method with the control set to 1.

### Western blot

Total protein was extracted with the M-Per Mammalian Protein Extraction Reagent (Pierce Biotechnology, MA, USA). The proteins (60 μg) were separated by SDS-PAGE and transferred to polyvinylidene difluoride membranes for immunoblots with antibodies against iASPP (sc-81297; Santa Cruz), p53 (sc-126; Santa Cruz), p21 (sc-397; Santa Cruz), Bax (sc-493; Santa Cruz), MMP-2 (#13132; Cell Signaling), MMP-9 (HPA001238; Sigma-Aldrich) or GAPDH (sc-47724; Santa Cruz). These primary antibodies were used at a dilution of 1:1000.

### Cell proliferation, migration, invasion assays

Cell proliferation was determined using a Cell Counting Kit-8 (Dojindo, Japan). Briefly, 5 × 10^3^ cells were plated in 96-well plates, incubated for 24 hours and then transfected with the iASPP siRNA/iASPP cDNA vectors, miR-124 mimics/inhibitors, and their respective controls. At 24, 48 and 72 hours, the absorbance of the cells was measured with a spectrophotometer at 450 nm. The transwell invasion and migration assays were performed as previously described^32^,^33^ with transfected cells seeded into the upper chamber (BD Biosciences, MA) with or without a Matrigel coating and DMEM/F12 with 10% FBS in the lower compartment acting as a chemoattractant. Cells were allowed to migrate for 12 and 24  hours in the migration and invasion assays, respectively. The non-motile cells were removed from the top, and the number of cells that had migrated or invaded was quantified by Giemsa staining. Relative migration and invasion activities are expressed as the fold-change over their respective controls.

### Flow cytometry analysis

In brief, CC cells were harvested and washed twice with cold PBS and resuspended in binding buffer containing Annexin V-FITC (Beckman Coulter Immunotech, Marseille, France) and subjected to PI staining incubation for 15 min at 4 °C in the dark. The stained cells were analyzed with flow cytometry within 30 min. The percentage of apoptotic cells was calculated using ModFit LT software (Verity Software House). Early apoptotic cells are Annexin V-positive and PI-negative (Annexin V-FITC^+^/PI^−^), whereas late apoptotic cells are Annexin V/PI-double-positive (Annexin V-FITC^+^/PI^+^).

### Caspase-Glo 3/7 assay

The caspase-3/7 activities were measured by using a Caspase-Glo 3/7 assay kit according to the manufacturer′s instructions (Promega, Madison, WI) as previously reported^34^. The luminescence was measured after 3 hours of incubation with the caspase substrate.

### Sphere formation assay

Single cell suspensions were suspended at a density of 5,000 cells/ml in serum-free DMEM/F12 medium, supplemented with N2 plus media supplement (Invitrogen, CA), 20 ng/ml epidermal growth factor (Invitrogen, CA), 20 ng/ml basic fibroblast growth factor (Invitrogen, CA), and 4 mg/ml heparin (Sigma-Aldrich, UK) and seeded into Ultra-Low Attachment 6-well plates (Corning Inc., Corning, NY). Fresh medium was added to each well every 3 days. The suspension cultures were continued for 14 days, and then the number of spheres larger than 50 μm was counted.

### Co-Immunoprecipitation assay and Western blot

The co-immunoprecipitation was performed using an ImmunoCruz^TM^ IP/WB Optima E System (Santa Cruz, UK), as previously described^35^. 1 mg whole cell lysates were pre-cleared with irrelevant rabbit IgG (Life Technologies) for 2 hours and subsequently incubated overnight with anti-iASPP antibody (sc-53864; Santa Cruz), whereas rabbit IgG was used as the negative control. The bound proteins were analyzed by using Western blotting with p53 antibody (sc-126; Santa Cruz).

### ChIP and qPCR analysis

To show that p53 protein directly interacts with the *p21*, *Bax* and *MMP-2* promoter regions, we performed a ChIP-qPCR analysis using a Pierce Agarose ChIP kit (Pierce, Thermo Scientific, Rockford, IL) according to the manufacturer′s protocol. The following antibodies were used for the immunoprecipitations: mouse monoclonal p53 antibody (sc-126; Santa Cruz) or unrelated rabbit IgG (Life Technologies) as a negative control. The immunoprecipitated DNA was reverse cross-linked, purified and analyzed by qPCR were using primers specific for the *p21*^8^, *Bax*^9^, *MMP-2*^10^ and *GAPDH*^11^ genomic loci. Results were expressed as the fold enrichment over the IgG control and further normalized to the *GAPDH* promoter.

### Transactivation assay

CC cells, seeded in 24-well plates, were transfected with one of the *p21*^12^, *Bax*^12^ or *MMP-2*^13^-luciferase promoter plasmids (100 ng) and the Renilla luciferase plasmid pRL-CMV (10 ng), together with (or without) p53 expression vector (50 ng) or p53 siRNA (6 nM), in the presence (or absence) of increasing amounts of iASPP expression vector (25 or 50 ng) or iASPP siRNA (3 or 6 nM), using Lipofectamine 2000 reagent (Invitrogen, CA). The pRL-CMV plasmid served as an internal control for normalizing the transfection efficiency. Luciferase activities were determined after 24 hours, using the Dual Luciferase assay kit (Promega, WI). Firefly luciferase activities were calculated relative to Renilla luciferase activities.

### 3′**-**UTR reporter assay

Human *iASPP* 3′-UTR cloned downstream of the firefly luciferase gene was purchased from OriGene Technologies (Rockville, MD). A mutation in the miR-124 seed-matching sequence was generated using a quick-change site-directed mutagenesis kit (Stratagene, CA). Cells were seeded in 24-well plates and transfected after 24 hours with wild-type or mutated firefly reporter vectors, Renilla reporter plasmid pRL-CMV for normalization, and miR-124 mimic or anti-miR-124 inhibitor or their negative controls (30 nM). Cells were collected 24 hours after transfection, and the firefly and Renilla luciferase activities were measured using a Dual Luciferase assay kit (Promega, WI). The firefly luciferase activity was normalized to the Renilla luciferase activity.

### Statistical analysis

The data are expressed as the mean ± SEM of at least three independent experiments performed in triplicate. If not specified otherwise, the experimental values are expressed as fold-changes normalized to their respective controls. Differences were analyzed using Student’s *t*-test or One-way ANOVA or chi-square (χ^2^) test, and results were considered statistically significant at *P *< 0.05. The differences between the cancer and normal tissues were analyzed using the Wilcoxon matched-pairs test. The log-rank test was used for survival analysis.

## Additional Information

**How to cite this article**: Dong, P. *et al*. Suppression of iASPP-dependent aggressiveness in cervical cancer through reversal of methylation silencing of microRNA-124. *Sci. Rep.* **6**, 35480; doi: 10.1038/srep35480 (2016).

## Supplementary Material

Supplementary Information
