# Supplementary material for: Suppression of iASPP-dependent aggressiveness in cervical cancer through reversal of methylation silencing of microRNA-124
Source: Sci Rep. 2016 Oct 21;6:35480. doi: 10.1038/srep35480 (PMC5073231; doi:10.1038/srep35480)
Supplement: Supplementary Information [file srep35480-s1.pdf]

Supplemental Information

Suppression of iASPP-dependent aggressiveness in cervical cancer through reversal of methylation silencing of microRNA-124

Peixin Dong[1, *], Ying Xiong[2, *], Hidemichi Watari[3], Sharon JB Hanley[1], Yosuke Konno[3], Kei Ihira[3], Fumihiko Suzuki[4], Takahiro Yamada[1], Masataka Kudo[3], Junming Yue[5] & Noriaki Sakuragi[1, 3]

[1]Department of Women's Health Educational System, Hokkaido University School of Medicine, Hokkaido University, N15, W7, Sapporo 0608638, Japan

[2]Department of Gynecology, State Key Laboratory of Oncology in South China, Sun Yat-sen University Cancer Center, Guangzhou 510060, P. R. China

[3]Department of Gynecology, Hokkaido University School of Medicine, Hokkaido University, N15, W7, Sapporo 0608638, Japan

[4]Department of Obstetrics and Gynecology, Tohoku University, Sendai 9808574, Japan

[5]Department of Pathology and Laboratory Medicine, University of Tennessee Health Science Center, USA; Center for Cancer Research, University of Tennessee Health Science Center, Memphis, TN, USA

[*]Equal contributors

Correspondence should be addressed to: P. D. (email: dpx1cn@gmail.com), H. W. (email: watarih@med.hokudai.ac.jp) and J. Y. (email: jyue@uthsc.edu)

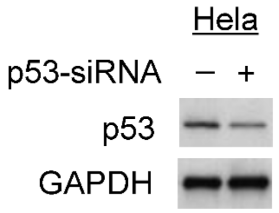

Supplementary Figure 1: Immunoblot of p53 protein expression in Hela cells after knockdown of p53.

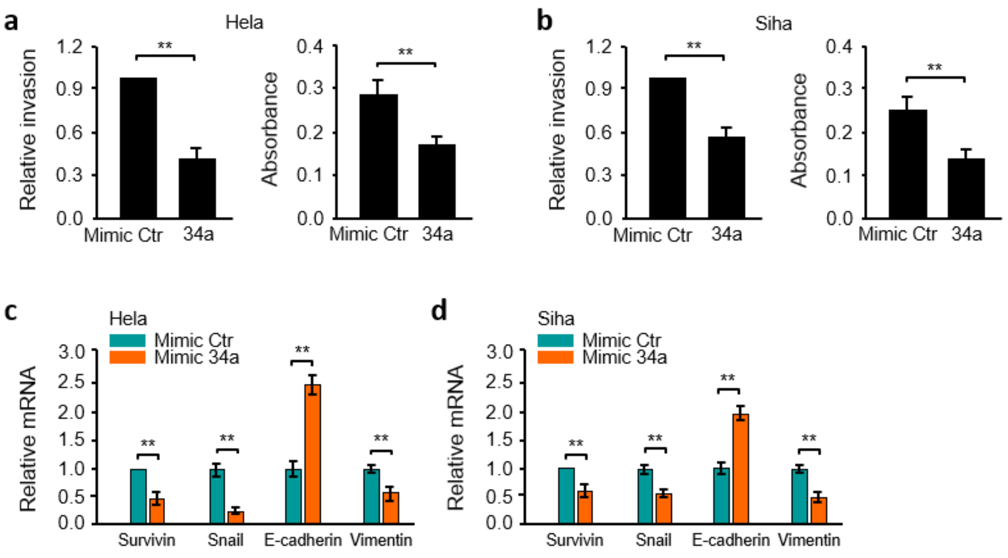

Supplementary Figure 2: miR-34a functions as a tumor suppressor in CC cells. Invasion (left) and proliferation (right) of Hela (**a**) and Siha (**b**) cells transfected with control or miR-34a mimic. qPCR analysis of the indicated mRNAs in Hela (**c**) and Siha (**d**) cells transfected with control or miR-34a mimic. The data are shown as the mean ± SEM; n = 3; **$P < 0.01$.
